# Gendered Sources of Distress and Resilience among Afghan Refugees in Northern California: A Cross-Sectional Study

**DOI:** 10.3390/ijerph14010025

**Published:** 2016-12-28

**Authors:** Carl Stempel, Nilofar Sami, Patrick Marius Koga, Qais Alemi, Valerie Smith, Aida Shirazi

**Affiliations:** 1 Department of Sociology and Social Services, California State University, East Bay, 25800 Carlos Bee Blvd., Hayward, CA 94542, USA; 2 Institute of Human Development, University of California, Berkeley, 1121 Tolman Hall #1690, Berkeley, CA 94720, USA; nilofarsami@gmail.com; 3 Department of Public Health Sciences, UCD School of Medicine, University of California, Davis, One Shields Avenue, Med Sci 1-C, Davis, CA 95616, USA; pmkoga@ucdavis.edu; 4 Department of Social Work & Social Ecology, School of Behavioral Health, Loma Linda University, 1898 Business Center Drive, San Bernardino, CA 92408, USA; qalemi@llu.edu; 5 Department of Health Sciences, California State University, East Bay, 25800 Carlos Bee Blvd., Hayward, CA 94542, USA; valeriejs2@gmail.com; 6 School of Public Health, Health Policy and Management, University of California Berkeley, 265 University, Hall, Berkeley, CA 94702, USA; shiraziaida@berkeley.edu

**Keywords:** refugee mental health, gender and mental health, Afghan, resettlement stressors, dissonant acculturation, gender ideology

## Abstract

Recent studies have emphasized the influence of resettlement factors on the mental health of refugees resettling in developed countries. However, little research has addressed gender differences in the nature and influence of resettlement stressors and sources of resilience. We address this gap in knowledge by investigating how gender moderates and mediates the influence of several sources of distress and resilience among 259 Afghan refugees residing in Northern California (USA). Gender moderated the effects of four factors on levels of distress. Intimate and extended family ties have little correlation with men’s distress levels, but are strongly associated with lower distress for women. English ability is positively associated with lower distress for women, but not men. In terms of gender ideology, traditionally oriented women and egalitarian men have lower levels of distress. And experiencing greater dissonant acculturation increases distress for men, but not women. The influence of gender interaction terms is substantial and patterns may reflect difficulty adapting to a different gender order. Future studies of similar populations should investigate gender differences in sources of distress and resilience, and efforts to assist new arrivals might inform them of changes in gender roles they may experience, and facilitate opportunities to renegotiate gender roles.

## 1. Introduction

In recent years a great deal of research has focused on the influence of resettlement factors on the mental health of refugees resettling in developed countries. However, little empirical work on refugee mental health has addressed the gendered nature of stressors and sources of resilience. This study seeks to address this gap in knowledge by investigating the role of gender in moderating and mediating the influence of various sources of distress and resilience among current and former Afghan refugees residing in Alameda County (California, USA).

Research has shown that refugees experience high levels of trauma and loss [1,2] and refugees resettling in developed countries are at greater risk of depressive and anxiety disorders, including PTSD, as compared to natives or labor migrants [3,4,5]. Recent research utilizing ecological or psycho-social perspectives acknowledges the importance of war traumas and trauma-based therapies, and adds a focus on the losses and stressful conditions refugees experience while seeking asylum and adjusting in a new society [6,7,8]. This focus is supported by a number of studies finding that, net of traumatic pre-migration and transit-related experiences, daily resettlement or post-migration stressors and sources of resilience explain a substantial part of the variation in current levels of distress in refugee populations [9,10,11].

Previous studies have found a variety of factors that negatively affect mental health, including but not limited to: acculturation [12] and cultural adjustment challenges (including language acquisition) [13]; loss of social support from one’s ethnic community [14]; family conflict [15]; unemployment and financial hardships [16]; legal migration status and conflict with immigration officials [17]; and, ethnic discrimination [18,19]. Conversely, factors supportive of higher resilience and positive mental health among conflict-affected populations include a sense of coherence, strong family and social networks (from same ethnic communities), coping, as well as religion and belief systems [20].

Findings from the studies described above parallel previous studies of Afghan refugees consolidated in a recent systematic review showing high rates of depression, PTSD, and psychosomatic symptoms as influenced by similar post-resettlement factors, with women showing higher vulnerability [21,22]. However, as mentioned, few researchers have investigated the gendered nature of stressors and sources of resilience [23], and we found no studies that addressed this issue for Afghans. This study seeks to address this gap in the literature using data from a cross-sectional survey of current and former Afghan refugees, developed through informants and preliminary qualitative interviews of Afghans residing in Alameda County, California.

Many of the resettlement stressors raised by informants and subjects in qualitative interviews during survey development were highly gendered. These included reports of family conflicts over women adopting non-traditional roles, suppression or marginalization of women who are “moving too fast” to expand their roles, status-loss among middle and upper class males who struggled to transfer their credentials and professional credibility in the USA, and intergenerational tensions over Afghan adolescents and young adults’ (especially daughters’) perceived excessive “Americanization” and disregard of Afghan culture, especially around dating and sexuality, much of which parallels Lipson and Omidian’s [24] ground-breaking research with Afghan refugees. Of course, many of these are sources of stress for other immigrant groups [25,26], but we believe they may be particularly influential for Afghans because of substantial differences between the gender and sexual orders of Afghanistan and the USA, and because of the increased vulnerability of Afghan refugees who have experienced significant traumas [27], have family members and friends in danger in Afghanistan, and who are distressed by the continuing wars and strife in Afghanistan [28].

Much work on gender and migration focuses on the disruption of “traditional” or “patriarchal” gender roles and balances of power faced by migrants entering host societies with liberalized or more egalitarian gender orders. Landmark studies caution against simplistic immigrant acculturation theories that model gender change as based on immigrants assimilating egalitarian gender roles or norms of host societies [29,30,31]. Hondagneu-Sotelo’s [30] study of Mexican-Americans migrants in Redwood City, CA found that gender role expansion for many men and women was caused by family stage migration patterns that separated spouses and pressured them to develop skills outside of their gender spheres. Kibria’s study [29] of Vietnamese refugees in Philadelphia emphasized gender differences in institutional opportunities in the host society that altered the gender balance of power within families and communities to increase women’s power, but that did *not* lead to the adoption of gender egalitarian beliefs (acculturation). Similarly, a recent study of Syrian refugees in Lebanon found high unemployment among the husbands and the expansion of women’s public and economic roles. Under these conditions, some women reported losing their sense of femininity and greater stress from an increased workload, while a few reported feeling empowered by their expanded roles. Some Syrian men reported shame about not providing for and protecting their families, and some informants reported increased angry outbursts, physical violence and abuse towards women and children by Syrian men unable to perform their roles [32]. In her study of transnational Mexican families Hirsch [31] highlighted generational changes in *both the sending and receiving communities* that contributed to the erosion of patriarchal sexual and intimate relationships between spouses and the growth of a more egalitarian regime of “trust” among younger generations in both locations.

These and other studies encourage researchers to avoid essentializing the gender orders of sending and receiving societies, focus on how gender and generational power relations are influenced by diverse conditions of migration and resettlement, be attuned to the complexity of acculturation processes, avoid one-sided cultural determinism, and recognize the influence of transnational ties and flows. In this study we seek to recognize the complexity of gendered influences on refugee resettlement by exploring ways gender mediates or moderates the influence of a range of potential stressors and sources of resilience on levels of distress that Afghans experience during resettlement in northern California.

Our analysis tests for the effects of several variables previously shown to influence distress levels of immigrant and refugee groups. These include perceived discrimination, employment status, non-family social support, English ability, civic engagement, intimate and extended family ties, dissonant acculturation, and gender ideology. We examine the influence of gender on these factors and expect to find that gender will moderate the influence of English ability, family ties, dissonant acculturation, and gender ideology. Additionally, we expect gender will moderately mediate the influence of employment and English ability on levels of distress.

### Hypotheses

#### Hypothesis 1: Women Will Report Higher Levels of Distress than Men

Across a wide range of social positions and conditions, women report higher levels of distress [33]. Previous work on refugees and Afghan refugees has found this same pattern [21,22].

#### Hypothesis 2: Gender Will Moderate the Effect of English Ability on Levels of Distress. English Ability Will Be More Strongly and Negatively Associated with Distress for Women than for Men

Beiser and Hou [34], found that for Southeast Asians, 10 years after relocating to Canada, low English ability was associated with depression for women but not men. They hypothesized that this reflects how English ability limits access to employment more for women than men (female-typed jobs more often require English competence) and that low English ability is associated with social isolation for women more than for men. We believe similar processes may be working for Afghan women in Alameda County, and add that social isolation and low English skills may be a self-reinforcing cycle for Afghan women more than men partly because of gender roles and power dynamics that encourage some women to stay in the home.

#### Hypothesis 3: Gender Will Moderate the Effect of Gender Ideology on Levels of Distress Such That Non-Traditional Women and Traditional Men Will Experience Higher Levels of Distress

Das Gupta [26] found that among high SES Asian Indian immigrants in the northeastern USA, both men and women with egalitarian views on gender had higher levels of anxiety. She suggested this may be because they were being marginalized by co-ethnics for threatening a strong cultural ideal of Indian women as chaste, pure, and family oriented. Our expectations that gender moderates the effect of gender ideology (traditional v. egalitarian views on gender roles) and family ties are informed by this study, but also by research on the gender order in Afghanistan and gender tensions among Afghans in the USA, and qualitative interviews about Afghan men’s difficulties enacting traditional gender roles in the USA.

It is important to note that solid majorities of Afghan women and men in northern California support more equal gender roles and expanding women’s roles. Many first-generation Afghans in the USA lived in Kabul prior to leaving and identify with urban modernity, including greater gender equality. In qualitative interviews, some discussed fleeing Afghanistan to escape the social repression of women during Taliban rule. We believe the desire for gender equality registered in our survey is genuine, but that it coexists with pockets of gender traditionalism and substantial gender segregation organizationally, occupationally, and domestically, and with sanctions against women who violate these boundaries [35]. We further hypothesize that egalitarian ideals coexist with more patriarchal “bottoms” to their gender ideologies [36] that reflect a gender order in Afghanistan which often include stronger gender segregation (e.g., “purdah” practiced by many Pashtun’s to prevent women from having contact with non-family males) and male authority, including an emphasis on male moral authority [37,38,39]. Thus, we hypothesize that many egalitarian Afghan women face resistance from partners, other family members, and community members that limit them from enacting egalitarian role identities, contributing to higher levels of distress. Traditionally oriented women may be better able to enact gender role identities that are consistent with their beliefs, and for some women (e.g., employed, active in public spheres) adapting a traditional gender ideology may be part of a “transitional” strategy that reduces stressful conflicts associated with more egalitarian practices [29,36,40].

Conversely, we hypothesize that men with traditional gender ideologies are likely to be more distressed because they are less able to enact gender role identities that fit their ideology while more egalitarian men may be less distressed because they have downgraded the importance of enacting provider or moral authority roles. Fully enacting provider roles has been challenging for many first generation Afghan men in our sample. Many Afghan men have had to accept less skilled, lower status, and lower paying work than they were qualified or were on track for in Afghanistan and/or have struggled, often with limited success, to get their credentials recognized or supplemented in their profession. Of the 104 Afghan men age 65 or under in this study, 11.6% were unemployed, 15.4% were working less than 25 h a week, 4.8% were retired, 11.5% were disabled, and 1.9% were ‘keeping house’. Of the 67 men age 65 and under who were employed, 47.8% said their job did not allow them to use their training, experience, and abilities and only 35.8% said their job utilized their training and skills. Thus, we expect that both employed and unemployed men benefit from an egalitarian gender ideology.

#### Hypotheses 4: Gender Will Moderate the Effect of Family Ties on Levels of Distress Such That Family Ties Will Be a Stronger Source of Resilience for Women than for Men and 4a: For Men, Extended Family Ties Will Be Least Buffering or a Source of Distress

Our expectation on gender moderating the influence of family ties is closely related to the struggles Afghan men have faced enacting traditional provider, protector, and moral authority roles and to informants’ reports of conflicts and stressors related to extended family ties. As just mentioned, many Afghan men struggle to enact provider roles, and they may experience conflicts with wives who are expanding their gender roles, and/or experience tensions with their children related to dissonant acculturation that they interpret as challenging their moral authority. Under these conditions family ties may not serve as a buffer for some men, or they may even be stressful reminders of their “failures” and frustrations in the occupational sphere or in carrying out their culturally important roles as moral leaders in the family [37,39].

Supporting our hypothesis, Dalgard and Thapa [41] found that among “non-Western immigrants” (from the Middle East, Africa, the Indian subcontinent, and the rest of Asia) to Norway, marriage was protective for women, but not for men. They also reported that non-Western immigrants in Norway have very high rates of unemployment (34%), perhaps reflecting conditions where men in this population had difficulty performing expected roles.

In addition, some young adult informants reported high levels of conflict between fathers and uncles and that in the jostling for status within extended families, some men are reminded of their failure to perform expected roles, making extended family interactions stressful. Thus, we hypothesize that extended family ties may even be a source of distress for men.

Conversely, we expect that family ties will have a robust buffering effect for Afghan women. Possessing nuclear and extended family ties allows women to enact traditional roles that continue to be highly valued in this Afghan community. Family ties provide them opportunities to enact important gender role identities without reinforcing the shame that some men may experience. To be clear, we do *not* expect that women in ‘traditional’ homemaker roles will have the lowest levels of distress. On the contrary, we expect that employment is a robust source of resilience for women and men.

#### Hypothesis 5: Gender Will Moderate the Effect of Dissonant Acculturation on Levels of Distress. Dissonant Acculturation Will Be More Strongly and Negatively Associated with Distress for Men than for Women

Portes and Rumbaut’s [42] conception of dissonant acculturation emphasizes acculturation gaps between parents and children as potential stressors. We did not find studies that identified gender differences in the influence of dissonant acculturation on distress. Nevertheless, we expect dissonant acculturation to be associated with greater distress for Afghan men because an acculturation gap constitutes men’s failure to protect the virtue of their children (especially daughters) and to enforce cultural/moral standards in their children, both salient father role identities for many Afghan men. This may have been reflected in separate focus groups of Afghan men and women when we raised issues of their children becoming too “Americanized”. Both men and women complained strongly about losing control of their children, but in contrast to the women the men linked this more to a sense of cultural loss. At one point in the men’s interview they collectively agreed they were “dinosaurs” and in explaining what this meant several men talked of cultural extinction and loss that was missing from women’s complaints. We hypothesize that expressions of cultural loss associated with dissonant acculturation reflect men’s loss of status and moral authority. It may also be that women are less distressed by dissonant acculturation because they recognize that the acculturation gap is a consequence of living in a society with opportunities for role expansion that they value.

#### Hypothesis 6: Gender Will Partially Mediate Employment-Distress and English Ability-Distress Relationships

In addition to testing for the moderating effects of gender we will run tests of its mediating effects. We expect women will have lower rates of employment and English competence, and that gender, English ability, and employment will be associated with distress. Thus, we expect gender to partially mediate the employment-distress and English ability-distress relationships.

## 2. Materials and Methods

### 2.1. Participants and Procedures

Inclusion criteria for this cross-sectional study entailed being of Afghan ancestry, originally resettled in the USA as refugee or under conditions of duress, and being an adult over the age of 18. Using the ancestry and birthplace questions in the 2005–2009 American Community Survey to match the time of our survey we estimate that 88,556 people of Afghan ancestry lived in the U.S at the time of our survey of which approximately 40% resided in California. Of the Afghans in California, 55.1% live in five contiguous counties running from the eastern San Francisco Bay Area to the Central Valley (Alameda, Contra Costa, Santa Clara, Sacramento, San Joaquin). Our sample was taken from Alameda County, which has by far the largest Afghan population of those five counties [43].

Recruitment took place between November 2007 and May 2008 using a combination of non-random and random sampling techniques through eight organizations or programs serving the local Afghan community. These included two mosques whose members were mostly Afghan, a cultural organization for a particular ethnic group and an organization that aided recent arrivals working to establish residency or citizenship. The other four programs/organizations provided opportunities for socialization, social support, and various services such as health screening, assistance navigating social service or medical bureaucracies, rent and food assistance, and referrals to mental health services. Participants were recruited by representatives of the different organizations. If the organization had a basis for developing a sampling frame we created the list and selected participants at random. Slightly over half (50.2%) of the sample was created this way. In some instances, this was not possible so we sampled purposively to create a representative gender and age balance. One of the eight organizations was added near the end to improve ethnic balance. We purposively selected organizations to insure each wave of migration was well represented. The research, survey design, and signed consent process were approved by the Institutional Review Board at the lead author’s university.

The survey of primarily fixed-choice questions was constructed by the first two authors aided by a team of Afghan informants and community leaders, and from insights gained in our preliminary qualitative research with Afghan refugees and their adult children as well as Afghan and non-Afghan social service providers exploring the social, cultural, medical, and mental health needs and challenges of Afghans in Alameda County. Many measures were developed or adapted based on this research and in some instances topics and standard measures (e.g., family conflict) were avoided because informants indicated responses would not be valid. Informants encouraged us to limit the number of questions on traumatic experiences causing us to limit somewhat items on pre-resettlement traumas.

After piloting an English version of the survey with Afghans fluent in English, two Dari translations and a Dari-in-English transliteration were produced by two bilingual Afghan educators and a graduate student with training in social science research methods. Differences were reconciled in conference with the lead author, a bilingual 2nd generation researcher with social science training, and two bilingual 1st generation informants (one with social science training) to produce exactly matching Dari and Dari-in-English versions. The latter version was used by interviewers fluent but not literate in Dari. This survey was pre-tested by conducting 20 interviews leading to final changes based on feedback from interviewees and interviewers. The survey was then computerized using Computer Aided Personal Interviewing software to improve coding accuracy and reliability of skip sequences [44]. Interviewers were matched by gender, and, when deemed necessary, by ethnic group (e.g., Tajik, Pashtun).

### 2.2. Measures

#### 2.2.1. Dependent Variable

The dependent variable in our analyses is the 24-item Talbieh Brief Distress Inventory (TBDI) which was designed and validated as a general measure of distress among immigrants [45]. TBDI draws 11 questions from the Psychiatric Epidemiology Research Interview Demoralization Scale and 13 items from the Brief Symptom Inventory that include items related to obsessiveness, hostility, sensitiveness, depression, anxiety, and paranoid ideation. Items are based on a one-month recall period where respondents are asked to indicate the degree of discomfort caused by each item with response choices ranging from 0 = “not at all” to 4 = “extremely”. Mean scores range from 0–4 with higher scores indicative of higher severity of distress. For our sample TBDI demonstrated strong internal reliability (Cronbach’s α = 0.960).

While we acknowledge that the TBDI has not been used as widely as measures developed for, or validated with refugee populations, items within the TBDI indeed mirror items from the HSCL’s anxiety and depression subscales. Also, the choice to use this measure provides a broader account of distress that is not solely limited to anxiety and depression given its subscales that tap into symptoms associated with hostility, paranoid ideation, etc., that likely manifest in traumatized populations. The scale demonstrated good face validity when presented to key informants in the Alameda County area, and we tested the scale in our pilot and pretest before fully implementing it.

#### 2.2.2. Independent Variables

The primary moderator and mediator variable is gender which was recorded as a binary variable in this study (1 = male, 0 = female). Core control variables were age in years, education, employment status, and English ability. Education is a four category variable measuring the highest level of education achieved in Afghanistan, the USA, or some other country (ranging from 0 = “less than high school” to 3 = “4-year college degree or higher”). Employment status is a binary variable (1 = currently employed part-time or full-time, 0 = not employed). A small number of respondents who were attending school (*n* = 11) were counted as employed for this variable. English ability is the mean score of three items on self-rated ability in speaking, writing, and reading English (ranging from 0 = “Not at all” to 4 = “Very fluent”). The English ability scale demonstrated excellent reliability in this sample (Cronbach’s α = 0.973).

Other independent variables included perceived discrimination, civic engagement, dissonant acculturation, family ties, non-family support, pre-resettlement traumas, and gender ideology. *Perceived discrimination* is the mean score of four equally weighted items including two standard questions about personal experiences of discrimination, one asking if they experienced discrimination in any of several various contexts and the other on perception of how fairly Afghan job-seekers are treated. Two other items were developed through preliminary research to focus on experiences and perceived threats after the events of 9/11. Perceived discrimination has a Cronbach’s α of 0.557. While somewhat low, it is acceptable because different dimensions of discrimination were measured.

*Family ties* is a summated index of three yes/no items: Is the respondent married and living with his/her spouse? Is one or more other family members living in their household? Do they see 10 or more other family members (not in household) on at least a monthly basis (extended family ties)? Cronbach’s α for this variable is a very low 0.189. Nevertheless, combining these items is justified because the index captures different intimate and extended family ties that are likely to provide substantial emotional or practical support and/or opportunities to enact gender role identities. We thus treat this variable as multidimensional and conducted additional tests on the influence of both extended and intimate family ties. *Non-family support* is a single binary item asking it the respondent has a friend he/she can rely on when in need of help.

*Dissonant acculturation* is a summated index made up of four items. It includes two questions on dating that, based on interviews of young adult Afghans, reflect important acculturation gaps; a question on conflict with their children over them becoming too “Americanized”; and a question on how worried they are that their children will fail to maintain their Afghan culture and identity. The first dating question asked if it is ok for Afghan children in high school to go on mixed-gender group dates and the second question asks if it is ok for Afghan children to date people of the opposite sex after completing high school. USA dating practices are particularly distressing for many Afghan parents and many are aware that Afghan youth and young adults often hide their intimate relationships and premarital sexual intimacy from parents. The first three items are binary items and the last one on how much they worried that their children will fail to maintain their Afghan culture and identity was coded 0 = not worried, 1 = somewhat worried, 2 = worried a lot. We effectively weighted this item double the other three items because it is a more global measure that we felt deserved more weight. Two of the other items were on the same issue (dating), and we wanted to weigh the “conflict over Americanization” item less because of modest validity concerns about respondents disclosing family conflicts. Cronbach’s α was a modest 0.526 for this index because it includes three somewhat separate dimensions: views on dating that conflict with USA culture, general fear of cultural loss, and conflict with child over their Americanization.

*Pre-resettlement trauma* is a summated index of 11 “yes/no” items on respondents’ traumatic experiences encountered in Afghanistan or while fleeing. These included items such as, “Were any close friends or family members killed or missing during the wars or coups?” “Did you witness someone being killed or seriously injured?” “Was your life threatened during wars and coups in Afghanistan?” and “Did you live in a refugee camp after fleeing Afghanistan?” Cronbach’s α = 0.749 for this index.

*Civic engagement* is a summated index of four yes/no items asking respondents if they are a USA citizen, if they voted in the 2006 election, if they volunteered in an organization serving Afghans in the past year, and if they volunteered in another civic organization in the past year (not serving Afghans). Cronbach’s α = 0.541 for this variable.

*Gender ideology* is a single binary item from NORC’s General Social Survey that informants said would be well understood and would capture salient ideals about gender roles. The respondent is asked which type of marriage they would prefer and the two choices describe a traditional division of labor where the husband provides household income and the wife cares for the home and family and an egalitarian marriage where these responsibilities are shared equally. Those who selected the traditional marriage description were coded 1 and the others (including a few who volunteered ‘not sure’) were coded 0.

### 2.3. Data Analysis

We used SPSS version 24.0 (IBM, Armonk, NY, USA) for all data analysis. We handled missing data by replacing with means scores for respondents who answered at least 21 of 24 TBDI items and three of four perceived discrimination items, dropping those without 21 of 24 or three or four, respectively, from the analysis. For other variables cases were dropped for missing items, except we imputed values for two of the 11 items for 48 cases in pre-resettlement traumas (missing due to interviewer or computer error) using predicted values from models that regressed the item score on the remaining trauma items, gender, age, employment status, and education. We believe this solution for the missing pre-resettlement items only slightly affected the results because there was comparatively little variation in these two items. Almost seven in eight (87.1%) had experienced “life threatening situations”, while less than one in seven (13.9%) were “captured, imprisoned, or held hostage”. Pearson’s *r* between the 9-item index and the 11 item index is 0.987. When it made analytical sense, we treated “don’t know” responses as “no”. For example, two respondents who said they “don’t know” if they have a close friend they can rely on were treated as not having a close friend. The sample size for regressions ranged from 241 to 250 (We ran the ten regressions in Tables 2 and 3 with pre-resettlement trauma, discrimination, TBDI variables that excluded cases with any missing values. This reduced the sample size significantly, ranging from 185 to 241. The pattern of significant relationships remained the same with a few exceptions. Overall, our interpretations remain the same. Employment in Model 1 changed from marginally significant to significant at *p* < 0.05 (*p* = 0.049). Gender in Model 6 changed from *p* < 0.05 to *p* < 0.01. In Model 7 dissonant acculturation went from marginally to non-significant (*p* = 0.109). In Model 8 gender changed from significant at *p* < 0.05 to marginally significant (*p* = 0.069). The two most important changes were in Models 9 and 10. In Model 9 English × gender went from significant at *p* < 0.05 to marginally significant (*p* = 0.053). In Model 10, pre-resettlement trauma explained substantially more variance. Adding the version of this variable without imputed missing values for two items explained an additional 9.2% of the variance compared to 6.8% of added explained variance reported in Table 3. The R^2^*_adjusted_* for Model 10 also increased from 42.1% to 43.3%. When added to this model, pre-resettlement trauma × gender was not significant at *p* < 0.10. We also ran Model 10 with a nine item pre-settlement trauma index that dropped the two items with a large number of missing cases and the additional explained variance was also higher (8.3%) than reported Model 10 in Table 3. Thus, it is likely that we modestly underestimated the influence of pre-settlement trauma in the reported Model 10.).

**Figure 1:**
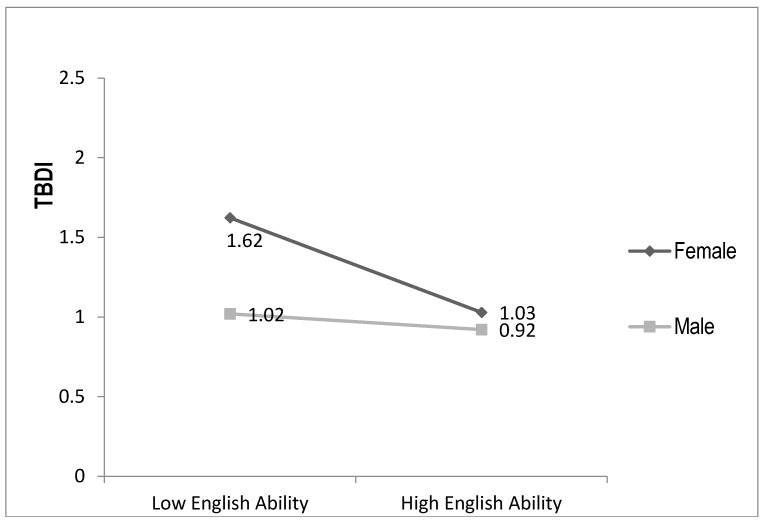
English by Gender Explaining Distress.

**Figure 2:**
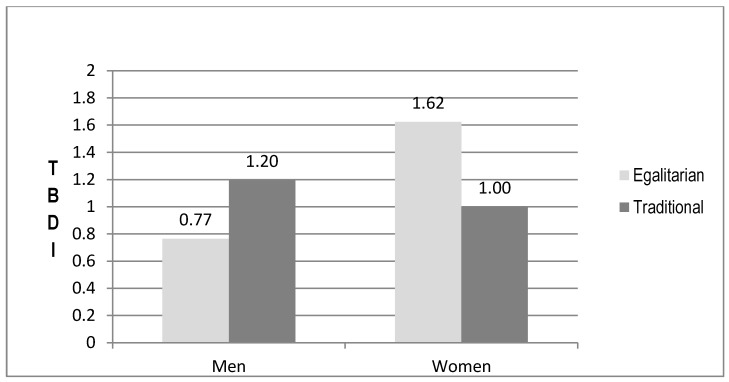
Traditional Gender Ideology by Gender Explaining Distress.

**Figure 3:**
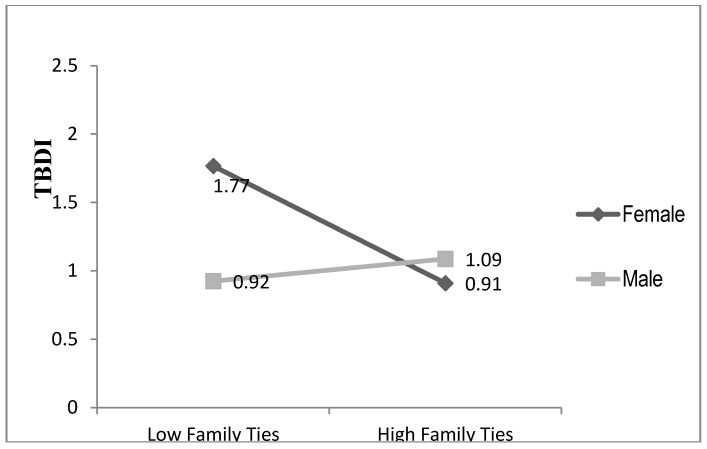
Family Ties by Gender Explaining Distress.

**Figure 4:**
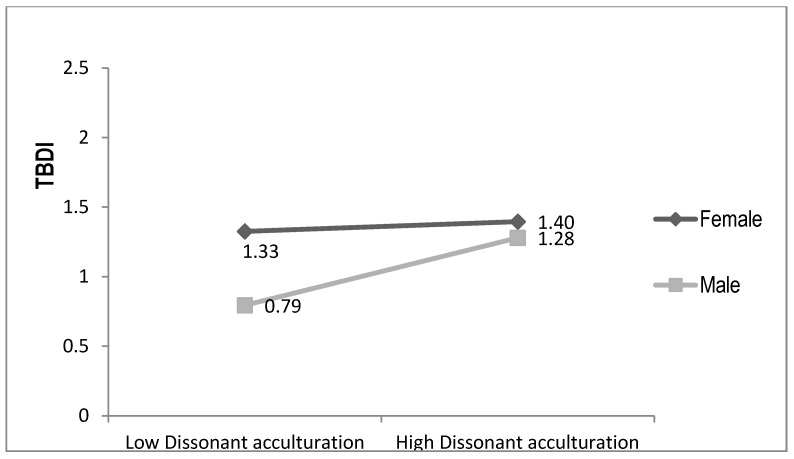
Dissonant Acculturation by Gender Explaining Distress.

Pearson, Spearman, and point-biserial correlations, were used to examine relationships between continuous, ordinal, and categorical independent variables and the dependent variable (TBDI). To test our hypotheses, stepwise multiple regression analyses explaining TBDI scores were used, consisting of 10 ordinary least squares (OLS) regression models organized hierarchically. In Step 1 we entered four socio-demographic variables as core controls; Step 2 added gender to assess its independent influence; and Step 3 added six key explanatory variables, thus constituting our full model of resettlement factors without gender interactions. Steps 4–7 each add to Model 3 one gender interaction term (e.g., English ability × gender in Model 4). We report only interaction terms significant at *p* < 0.10. We relaxed the *p* < 0.05 standard to *p* < 0.10 for interactions because of concern about Type II errors with our relatively small sample size and to identify possible moderators for future studies. Each of these interactions are plotted or graphed in Figure 1, Figure 2, Figure 3 and Figure 4. Model 8 drops two non-significant independent variables from Model 3 (core controls are kept regardless of significance levels) and Model 9 then adds all (three) of the statistically significant interaction terms to Model 8. Model 9 is thus our most parsimonious full model focusing on resettlement stressors and sources of resilience. Model 10 adds pre-resettlement traumas to create our full parsimonious model.

## 3. Results

### 3.1. Socio-Demographic Characteristics

Table 1 reports percentages for categorical variables and means, standard deviations and ranges for continuous variables. A sample of 259 Afghans participated in this study. Our sample was on average 48.75 (SD = 15.86) years of age and slightly more likely to be female (51.4%) with a majority possessing a high school diploma (29%) or lower (31%), and 25.1% holding a 4-year college degree or higher. Overall, 41.3% were employed (28.5% of women and 69.5% of men age 65 and younger), with moderate levels of English ability (M = 2.03, SD = 1.29), and year of arrival ranging from 1979 to 2008 (M = 1993, SD = 8.06). Average TBDI scores indicate that distress occurs at low to moderately high levels (M = 1.21, SD = 0.99).

### 3.2. Bivariate Analyses

The far right column of Table 1 reports Pearson correlations, Spearman’s rho, or point-biserial correlations and significance levels between the independent variables and the dependent variable (TBDI). With the exception of the ‘year of arrival’ variable, the core controls (age, education, employment, and English ability) have bivariate relationships with TBDI that are significant at *p* < 0.05 (two-tailed). Being younger, highly educated, employed, and possessing greater English competence are associated with lower levels of distress. Among the other explanatory variables English ability is negatively associated with TBDI scores and perceived discrimination, dissonant acculturation, and pre-resettlement traumas are positively associated with TBDI at *p* < 0.05.

### 3.3. Multivariate Analyses

#### 3.3.1. Hypothesis 1: Effect of Gender on TBDI

Moving to our regressions, in Model 1 (Table 2) we see that the four controls explain 8.2% of the variance (adjusted R^2^), with English ability (*p* < 0.05) and employment status (*p* < 0.10) both negatively associated with distress. Model 2 adds gender, which explains an additional 1.8% of the variance with men having lower levels of distress than women (*p* < 0.05). This finding supports hypothesis 1 that women report higher levels of distress.

Model 3 adds the remaining six independent variables, which combined explain another 15.2% of the variance. Having more family ties lowers distress levels, while experiencing discrimination and dissonant acculturation increase levels of distress. Based on standardized betas, discrimination and family ties are the strongest factors influencing distress levels. With these additional independent variables gender continues to be significantly related to TBDI (*p* < 0.01), further confirming hypothesis 1.

#### 3.3.2. Hypothesis 2: Gender Moderates Effect of English Ability on TBDI

We then, in separate models, added to Model 3 a gender interaction term one at a time for employment, English ability, discrimination, family ties, non-family support, civic engagement, dissonant acculturation, traditional gender ideology, and pre-resettlement trauma. Models 4 through 7 report the results of the four interaction terms that were significant at *p* < 0.10. The gender interaction terms for employment, discrimination, non-family support, civic engagement, and pre-resettlement traumas were not significant.

Model 4 shows that, with control and independent variables, the English ability × gender interaction is significant at *p* < 0.05 explaining an additional 1.6% of the variance in TBDI. Figure 1 plots this interaction, showing that English ability lowers distress levels for women more than for men, supporting hypothesis 2. Moving from low to high English ability reduces women’s predicted TBDI scores nearly 6/10 of a point on this four-point index, while for men increasing English ability only decreases predicted TBDI scores by 1/10 of a point.

#### 3.3.3. Hypothesis 3: Gender Moderates Effect of Gender Ideology on TBDI

Model 7 (Table 3) shows that the traditional gender ideology × gender interaction is significant at *p* < 0.001. Adding this interaction term explains a substantial additional 6.6% of the variance. Figure 2 graphs this interaction showing that traditional men have higher levels of distress than egalitarian ones, while the reverse is true for women. With controls, more egalitarian women’s TBDI scores are over 6/10 of a point higher than scores for traditional women. Traditional men’s TBDI scores are over 4/10 of a point higher than their more egalitarian counterparts. This pattern strongly supports hypothesis 5.

#### 3.3.4. Hypotheses 4 and 4a: Gender Moderates the Effect of Family Ties on TBDI; Extended Family Ties Are Least Buffering or a Source of Distress for Men

Model 5 shows that the family ties × gender interaction is significant at *p* < 0.001. Adding this interaction term explains an additional 5.4% of the variance. Figure 3 plots this interaction, showing that increasing family ties substantially lowers predicted distress levels for women, while for men more family ties increases predicted distress levels slightly. Moving from low to high family ties reduces women’s predicted TBDI scores over 8/10 of a point, but increases men’s predicted TBDI scores by over 1/10 of a point. This pattern strongly supports Hypothesis 3.

The family ties variable includes both intimate (marriage, other family members living in the household) and extended family ties. We tested which of these was most influential in this gender interaction. Separate regressions and plots for intimate and extended family ties (not shown) found that for men extended family ties actually *increase* predicted TBDI scores by just over 4/10 of a point, while intimate family ties have no influence on their distress levels. This supports hypothesis 4a that extended family ties would be most stressful for men. For women, intimate family ties reduce women’s distress levels the most, but extended family ties also lower their TBDI scores.

#### 3.3.5. Hypothesis 5: Gender Moderates the Effect of Dissonant Acculturation on TBDI

Model 6 shows that, as expected, the dissonant acculturation × gender interaction is marginally significant at *p* < 0.10 (*p* = 0.071). In Figure 4 we see that moving from lowest to highest levels of dissonant acculturation increases men’s predicted TBDI scores by nearly 5/10 of a point, while women’s increases just under 1/10 of a point. This finding fits the expected pattern, but only marginally supports Hypothesis 4.

Model 8 presents our most parsimonious model of resettlement factors that does not include the gender interactions. Model 8 removes the non-significant “non-family support” and “civic engagement” variables from Model 3 while explaining the same amount of variance in TBDI as Model 3 (R^2^*_adjusted_* = 23.5%).

Model 9 shows that adding three gender interaction terms (English × gender, family ties × gender, and traditional gender ideology by gender) explains an additional and *quite substantial* 12.1% of the variance in TBDI. This suggests that gendered aspects of resettlement factors are quite influential in explaining levels of distress among Afghan refugees in Alameda County. We planned on including in Model 9 gender interaction terms that were significant at *p* < 0.10. When included with the other gender interactions, dissonant acculturation × gender was not significant at *p* < 0.10, while the other three gender interactions are significant at *p* < 0.05 or better. Exploratory regressions that added gender interaction terms incrementally to models with dissonant acculturation suggest that the influence of gender ideology × gender most overlaps with dissonant acculturation × gender. Model 9 is our most parsimonious model relying on resettlement factors to explain distress. It explains over 1/3 of the variance in distress levels in this population (R^2^*_adjusted_* = 35.3%).

Model 10 adds “pre-resettlement trauma” to Model 9. It shows that with the other control and independent variables and interaction terms, pre-resettlement trauma explains an additional and substantial 6.8% of the variance in TBDI scores. With the exceptions of dissonant acculturation and English ability, whose influence declines by about 1/3 in both instances, adding pre-settlement trauma changes little in the other factors’ relationships with TBDI. It is noteworthy and worthy of further investigation that the resettlement factors most collinear with pre-resettlement traumas in explaining distress levels are acculturation variables. With pre-resettlement trauma included, Model 10 explains 42.1% of the variance in levels of distress (adjusted R^2^).

#### 3.3.6. Hypothesis 6: Gender Mediates the Effects of Employment Status and English Ability on TBDI

We also conducted tests of the mediating role of gender in relationships between independent variables and levels of distress, including employment status, English ability, civic engagement, dissonant acculturation, traditional gender ideology, family ties, and non-family support. For each test variable, we conducted OLS hierarchical regressions explaining distress, first including the test variable with the four controls, not including gender (three controls for employment and English ability). We then added gender and compared the unstandardized beta for the test variable in the two models. If the beta was reduced significantly we tested to see if the test, control (gender), and dependent variables had significant associations (*p* < 0.05) with each other. Gender did not strongly reduce any test variables’ relationships with distress, but gender did moderately reduce the regression coefficient for the employment-TBDI relationship by 38% (−0.329 to −0.204). As we saw in Table 1, gender and employment are both associated with distress (*p* < 0.001), and in addition men were much more likely to be employed than women (*p <* 0.001). Controlling for gender did not change any other variables’ regression coefficients more than 10%, including English ability, which was contrary to our expectations. Thus, overall, gender does not play a substantial role in mediating the effects of the stressors and sources of resilience for Afghans in this study, but does modestly mediate the effects of employment.

In summary, Hypotheses 1 that women will report higher levels of distress is confirmed, and Hypotheses 2–5 on the moderating effects of gender on English ability, family ties, dissonant acculturation, and gender ideology were supported by our tests, although the support for dissonant acculturation × gender was marginal. Our hypotheses on the mediating effects of gender were modestly confirmed for employment, but not for English ability.

## 4. Discussion

A strong body of research has emphasized the gender structuring of migration experience and the restructuring of gender relations through migration [46], but little research on resettlement stressors and mental health of refugees has used this gender lens. To address this gap in the literature we examined the moderating and mediating effects of gender on various sources of distress among a cross-sectional sample of Afghans residing in Alameda County, California. For three variables (family ties, English ability, and gender ideology) gender significantly and robustly moderates levels of distress, and for a fourth variable (dissonant acculturation) the gender interaction was marginally significant. Our data showed that having intimate and extended family ties has little correlation with men’s distress levels, but is strongly associated with lower levels of distress for women. Likewise, greater English ability is associated with lower distress for women, but not men. In terms of gender ideology, traditionally oriented women and egalitarian men have lower levels of distress. And experiencing greater dissonant acculturation increases distress for men, but not women.

The influence of gender as a moderating variable adds substantially to our understanding of the sources of distress and resilience for Afghans in our sample. Adding three gender interaction terms to our parsimonious model of direct effects explains a quite substantial additional 12.1% of the variance in distress levels. This suggests that gender moderating effects on sources of distress for refugee populations should be tested for more widely, perhaps especially where significant differences exist in the gender orders of sending and receiving countries. We also tested gender as a mediating variable for the same explanatory factors and found that gender modestly mediates the employment-distress relationship, but does not substantially mediate any of the other variables’ relationships with distress.

We believe the gender differences in the influence of resettlement stressors and sources of resilience reflect the effects of (often traumatized) migrants coming from a society with stronger gender segregation and more divided gender roles, navigating a society where these patterns are not sustainable [35,47]. Afghan men’s role identities are tied to protecting and providing for family members and to instilling/upholding moral standards within the family [48]. Thus, family ties may not have protective benefits for men when they are unable to enact important roles associated with those ties. Strikingly, we found that regular contact with a large extended family is positively associated with higher levels of distress for men. This is particularly concerning and worthy of further investigation.

Relatedly, dissonant acculturation may be associated with greater distress for men because a large acculturation gap constitutes their failure to enact role identities of protecting the virtue of their children (especially daughters) and to instill cultural standards in their children. While women in our sample actually score higher than men on the dissonant acculturation index (*p* < 0.05), an acculturation gap does not necessarily prevent women from enacting salient role identities.

The striking gender difference in the influence of gender ideology on distress also fits the “performing salient role identities” perspective [49,50]. It may be that men with traditional gender ideologies are less able to enact one or more gender role identities that fit their ideology while more egalitarian men have shifted their role identities to fit current conditions. This would reduce stress directly and indirectly by reducing their levels of dissonant acculturation. Supporting the latter, we found that, controlling for age, gender, education, and employment status, men’s predicted dissonant acculturation scores were 7/10 of a point higher for those with a traditional gender ideology than their non-traditional counterparts (*p* < 0.05).

Afghan women with an egalitarian gender ideology may experience greater frustration when partners, other family members, or community members prevent them from enacting egalitarian role identities or greater resistance and stigma when they do. For example, egalitarian women who are unemployed may experience more frustration with this position and egalitarian women who are employed may experience greater tension or conflict. Conversely, unemployed women with a traditional ideology may experience less frustration because their ideal is aligned with their current position, and employed women with a traditional ideology may experience less stress because they are effectively denying that they are challenging the dominant gender order [29,36]. Our analysis here is grounded in a view that a rather strong difference exists between the ‘top’ and the ‘bottom’ of the prevailing gender ideology in this Afghan community [36].

While egalitarian gender ideologies are associated with greater distress for women, we should recall that the egalitarian practice of paid employment is a source of resilience for women—the employment × gender interaction was not significant at *p* < 0.10. The relative autonomy of the influence of gender ideology from enacted roles was highlighted when we looked more closely at responses to the employment question. We found that women who selected “keeping house” had much higher levels of distress than those who were employed. Thus, we do not conclude that Afghan women in the USA are better off pursuing a strategy of gender traditionalism. Rather, our findings, taken together, suggest that the disruption of a traditional gender order among Afghans in exile has been the source of considerable distress for both men and women, and that finding ways to reduce gender related stressors may be an important key for addressing mental disorders among this population.

In this regard, we believe that Bellinger‘s [51] intervention built around guiding refugee couples to recognize changes in gender roles they are or will be experiencing, and then renegotiating roles is a promising approach. The focus on gender change through individual couple’s therapy can be expanded to include collective or community-based interventions. We believe it is crucial to develop such interventions in symmetrical dialogue with a wide range of community members, including many who have experienced gendered stressors personally. And a la Hirsch [31], interventions should recognize and incorporate changes and struggles over the gender order in Afghanistan and within Islam, and be grounded in a critique of the West vs. Islam/Afghan binary around gender that structures much discussion about Afghan and Muslim gender relations. We also believe that directly addressing masculinities as possible sources of stress among men may be helpful as El-Masri et al. [32] suggest.

Though not our focus, our findings point to a need for more diverse measures of acculturation in this line of research. Many studies use unidimensional measures such as host language ability as the sole or primary measure of acculturation, utilizing an implicit or explicit assimilationist model of acculturation [52]. Our findings on the gendered influence of English ability and the influence of dissonant acculturation warrant future research with Afghans and other refugee groups that explores the complexity of acculturation processes and echo Birman and Tran’s [12] study which provides a more variegated understanding of the nature and influence of acculturation processes.

We must also note the strength of perceived discrimination and pre-resettlement stressors in explaining current distress levels. In our full model (Model 10), not counting variables with interaction terms, these variables are the two most influential in explaining TBDI, based on standardized betas. The former highlights the importance of the mode of incorporation on refugee mental health. Not surprisingly, the stereotyping, exclusion, and surveillance faced by Afghans takes a mental health toll. We believe this finding suggests that Afghans may benefit from interventions similar to the one evaluated by Goodkind et al. [53] that tailor interventions and advocacy in careful dialogue with clients and provide opportunities for symmetric cultural exchanges with members of the host society. Future work should more closely examine the nexus of identity, discrimination, dissonant acculturation, and mental health among refugees, especially those who are Muslim or facing other strong ethnic/racial stereotypes or are arriving in a country that has intervened in their country [54]. The strong relationship of pre-resettlement stressors on TBDI also deserves exploration in future studies, especially given that about 60% of respondents arrived in the USA ten or more years ago.

### Limitations

Several limitations may have influenced the results obtained. First, although when possible we randomly selected from client or membership lists of the organizations helping us, it appears that our sample modestly overrepresents unemployed women, and less educated and older (ages 60–69), first generation Afghans (we compared our sample to the sample of adult Afghans in the 2005–2009 American Community Survey; details available upon request.).

Secondly, because of cost considerations we were not able develop a Pashto version of the survey. Although we achieved a good ethnic balance in our sample, we excluded, with one exception noted above, the small number of Afghans in Alameda County who speak Pashto, but not Dari. We believe this category of Afghans is primarily Afghan women with high gender traditionalism and perhaps greater social isolation.

Thirdly, because our study is cross-sectional in design we cannot attribute causation to the patterns uncovered. For example, we think that dissonant acculturation adds stress and feelings of loss to men’s lives that over time contributes to their levels of distress, but it is also possible that high levels of distress contribute to cultural reification that heightens feelings of distance and contributes to conflict with children over their failure to retain Afghan norms, thus increasing levels of dissonant acculturation. This illustrates the pressing need for panel studies following recently arrived refugees for a number of years measuring a range of sources of resilience and distress at different times.

Fourthly, we did not have adequate data to fully address gendered correlates of distress related to pre-resettlement vulnerabilities and sources of resilience, or selection processes influencing which Afghans were able to migrate to the USA For example, our measure of family ties includes the item “married, living with spouse” whose reference category for women is essentially female headed households. Thus, “gender × family ties” registers the influence of female headed households (e.g., widows, which greatly outnumber widowers) which may in turn reflect policies that select for women with high pre-migration levels of distress and vulnerabilities. An analysis of family ties comparing the influence of each item on women’s distress levels and the non-significant “gender × pre-resettlement traumas” regression term reported above suggest modest unmeasured gendered pre-resettlement sources of current distress levels. However, we cannot rule out these kinds of pre-resettlement effects on current levels of distress and thus, we may be overemphasizing explanations that focus on resettlement factors. The panel studies just mentioned should include upstream measures that more fully capture the vulnerabilities and sources of resilience of newly arriving refugees.

Another similar example involves addressing problems with conceptualizing English ability as a measure of “acculturation”. We challenged this view and argued for the importance of richer measures of acculturation, but may have retained its shadow by limiting English ability to a “resettlement” source of resilience. Yet many Afghan women (and men) in the study probably learned or began learning English in Afghanistan. For women in this study, English ability may in part register being traditional or non-traditional in Afghanistan, and the conditions and resources associated with this difference. Thus, our Beiser-inspired explanation of gender’s moderating effect on English ability may, again, be biased towards focusing on resettlement factors and explanations.

Finally, related to the previous concern, future research should be designed to better measure ways that gender influences levels of distress differently for refugees than for immigrants. For example, in the USA, refugees, but not most immigrants, are eligible for income supports that are greater for female headed households. These supports may provide refugee women beneficial levels of security not afforded to immigrants, but may also accentuate or lengthen the influence of patriarchal gender roles and boundaries from the sending country. For example, it is quite possible that the low rate of employment among women in our study fits this pattern. Thus, some part of the employment effect for women in our study may be a gendered influence of government supports available to refugee women. An important strength of our work lies in our dialogic approach to survey construction that relied heavily on informants and qualitative research to identify sources of distress and develop valid measures. Survey construction also benefitted from previous research on this population and refugee mental health that was richly informed by theories of gender relations and changing gender orders [35,46]. Future work should use more detailed measures for gender ideology and dissonant acculturation, and include measures of gender role identities and gendered practices that will allow a richer analysis of patterns of gender identities, beliefs, and practices and their relationships to distress.

## 5. Conclusions

This study examined the moderating and mediating effects of gender on various sources of distress and resilience among a cross-sectional sample of Afghans residing in the Alameda County, California. We found that gender moderated the effects of four factors on levels of distress (gender ideology, family ties, English ability, and dissonant acculturation), the influence of these gender interaction terms is substantial, and the pattern of relationships fits a population of traumatized migrants whose gender order has been disrupted moving from a society with more divided gender roles and greater paternal authority, adapting to a society where these patterns are not sustainable. Future research should investigate the relationships we identified in greater depth in a longitudinal and multimethod design that allows researchers to better understand the complex processes and patterns of acculturation, shifting role identities, and gender change among newly arriving Afghans and similar populations as they adapt to and integrate into their new society. Efforts to assist such populations adapt to their host societies should inform new arrivals of changes in the gender order they are or will be experiencing, and facilitate opportunities to reflect on these changes and renegotiate gender roles in ways that are grounded in their complex cultural influences and identities.

## Figures and Tables

**Table 1:** Descriptive statistics: dependent, control, independent variables, and bivariate relationships with dependent variable (*n* = 259).

Variables	Statistics				
	*n* (%)	$\bar{x}$	*Sd*	*Range*	*Correlation with TBDI*
Talbieh Distress Inventory (TBDI)	251	1.21	0.99	0–4	1.0 ***^,b^
Age in years	259	48.75	15.68	18–84	0.13 *^,b^
Gender					
Male	126 (48.6)				−0.25 ***^,d^
Female ^a^	133 (51.4)				
Education					
Less than high school	83 (32.0)	1.32	1.17	0–3	−0.22 **^,c^
High school diploma	75 (29.0)				
AA, some college, technical degree	36 (13.9)				
Bachelor’s Degree or Higher	65 (25.1)				
Employment status					
Employed (or student)	107 (41.3)				−0.27 ***^,d^
Not Employed ^a^	152 (58.7)				
English ability	259	2.03	1.29	0–4	−0.30 ***^,b^
Year Arrived in USA	257	1993.51	8.06	1979–2008	0.10 ^b^
Perceived Discrimination	247	0.68	0.56	0–2	0.27 ***^,b^
Family ties	259	1.68	0.81	0–3	−0.17 **^,b^
Non-family Support					
Yes	180 (69.5)				−0.03 ^d^
No ^a^	79 (30.5)				
Dissonant Acculturation	257	1.27	1.11	0–5	0.20 **^,b^
Gender Ideology					
Traditional	64 (24.8)				−0.02 ^d^
Not Traditional ^a^	194 (75.2)				
Civic engagement	259	1.27	1.11	0–4	−0.09 ^b^
Pre-resettlement traumas	254	5.57	2.44	0–11	0.49 ***^,b^

Notes: ^a^ = reference category; ^b^ = Pearson’s r; ^c^ = Spearman’s rho; ^d^ = Point-biserial r; * *p* < 0.05; ** *p* < 0.01; *** *p* < 0.001.

**Table 2:** Resettlement factors and factors moderated by gender explaining psychological distress (TBDI).

	Model 1		Model 2		Model 3		Model 4		Model 5	
	B (SE)	β	B (SE)	β	B (SE)	β	B (SE)	β	B (SE)	β
(Constant)	1.74 (0.27)		1.62 (0.28)		1.60 (0.31)		1.71 (0.32)		2.16 (0.330)	
Age	−0.00 (0.00)	−0.02	0.00 (0.01)	0.03	0.00 (0.01)	0.01	0.00 (0.01)	−0.00	−0.00 (0.00)	−0.05
Employed	−0.28 (0.15)	−0.14 ^‡^	−0.18 (0.16)	−0.09	−0.16 (0.15)	−0.08	−0.16 (0.15)	−0.08	−0.28 (0.14)	−0.14 ^‡^
Education	−0.04 (0.06)	−0.05	−0.02 (0.06)	−0.02	0.01 (0.06)	0.01	0.03 (0.06)	0.04	0.04 (0.06)	0.05
English Ability	−0.17 (0.06)	−0.19 *	−0.13 (0.06)	−0.18 *	−0.12 (0.05)	−0.17 *	−0.22 (0.07)	−0.32 **	−0.10 (0.05)	−0.13 ^‡^
Gender (Male) ^a^			−0.30 (0.14)	−0.15 *	−0.33 (0.13)	−0.17 **	−0.74 (0.22)	−0.38 **	−1.33 (0.27)	−0.69 ***
Discrimination					0.58 (0.11)	0.33 ***	0.56 (0.11)	0.32 ***	0.53 (0.10)	0.30 ***
Family ties					−0.28 (0.07)	−0.23 ***	−0.25 (0.07)	−0.21 ***	−0.50 (0.09)	−0.43 ***
Non-family Support					−0.11 (0.13)	−0.05	−0.13 (0.12)	−0.06	−0.12 (0.12)	−0.06
Civic Engagement					−0.02 (0.06)	−0.03	−0.01 (0.06)	−0.01	−0.03 (0.06)	−0.04
Dissonant Acculturation					0.08 (0.04)	0.13 *	0.09 (0.05)	0.14 *	0.07 (0.04)	0.11 ^‡^
Trad. Gender Ideology ^b^					−0.05 (0.13)	−0.02	−0.07 (0.13)	−0.03	−0.06 (0.13)	−0.03
English × Gender							0.19 (0.09)	0.30 *		
Family Ties × Gender									0.61 (0.14)	0.62 ***
R^2^	0.097 ***		0.115 ***		0.267 ***		0.282 ***		0.321 ***	
Adjusted R^2^	0.082 ***		0.097 ***		0.232 ***		0.245 ***		0.285 ***	
ΔR^2^			0.018 *		0.152 ***		0.016 * ^c^		0.054 *** ^c^	
*F-statistic* ΔR*^2^*			4.80		7.99		5.02 ^c^		18.42 ^c^	

Notes: ^‡^ *p* < 0.10; * *p* < 0.05; ** *p* < 0.01; *** *p* < 0.001 References: ^a^ = female; ^b^ = egalitarian or not sure. Explained variance: ^c^ = ΔR^2^ for adding interaction term to Model 3.

**Table 3:** Resettlement factors and factors moderated by gender explaining psychological distress (TBDI).

	Model 6		Model 7		Model 8		Model 9		Model 10	
	B (SE)	β	B (SE)	β	B (SE)	β	B (SE)	β	B (SE)	β
(Constant)	1.79 (0.33)		1.72 (0.30)		1.55 (0.30)		2.23 (0.30)		1.40 (0.33)	
Age	0.000 (0.01)	−0.001	0.00 (0.00)	0.01	0.00 (0.00)	0.00	−0.00 (0.00)	−0.05	−0.00 (0.00)	−0.04
Employed	−0.20 (0.15)	−0.100	−0.22 (0.14)	−0.11	−0.18 (0.14)	−0.09	−0.33 (0.14)	−0.17 *	−0.32 (0.13)	−0.16 *
Education	0.01 (0.056)	0.01	0.01 (0.05)	0.01	0.00 (0.06)	0.00	0.05 (0.05)	0.06	0.07 (0.05)	0.08
English Ability	−0.113 (0.054)	−0.16 *	−0.12 (0.05)	−0.17 *	−0.13 (0.05)	−0.18 *	−0.18 (0.06)	−0.26 **	−0.12 (0.06)	−0.17 ^‡^
Gender (Male) ^a^	−0.60 (0.204)	−0.31 *	−0.60 (0.14)	−0.31 ***	−0.31 (0.13)	−0.16 *	−1.78 (0.30)	−0.92 ***	−1.62 (0.29)	−0.83 ***
Discrimination	0.57 (0.107)	0.33 ***	0.62 (0.10)	0.35 ***	0.56 (0.10)	0.32 ***	0.53 (0.10)	0.30 ***	0.42 (0.09)	0.24 ***
Family ties	−0.28 (0.07)	−0.24 ***	−0.28 (0.07)	−0.24 ***	−0.28 (0.07)	−0.23 ***	−0.45 (0.08)	−0.38 ***	−0.41 (0.08)	−0.35 ***
Non-family Support	−0.10 (0.12)	−0.05	−0.086 (0.12)	−0.04						
Civic Engagement	−0.03 (0.06)	−0.03	−0.01 (0.06)	−0.01						
Dissonant Acculturation	0.02 (0.05)	0.04	0.07 (0.04)	0.11 ^‡^	0.08 (0.04)	0.13 *	0.06 (0.04)	0.10 ^‡^	0.04 (0.03)	0.07
Trad. Gender Ideology ^b^	−0.07 (0.13)	−0.03	−1.93 (0.41)	−0.85 ***	−0.07 (0.13)	−0.03	−0.70 (0.18)	−0.31 ***	−0.61 (0.17)	−0.27 ***
English × Gender							0.16 (0.08)	0.25 *	0.15 (0.08)	0.23 *
Family Ties × Gender	.						0.53 (0.14)	0.54 ***	0.49 (0.13)	0.50 ***
Disson. Accult. × Gender	0.12 (0.07)	0.190 ^‡^								
Gender Ideol. × Gender			1.21 (0.25)	0.89 ***			1.11 (0.24)	0.40 ***	0.99 (0.23)	0.36 ***
Pre-resettlement Trauma									0.12 (0.02)	0.30 ***
R^2^	0.277 ***		0.333 ***		0.264 ***		0.385 ***		0.452 ***	
Adjusted R^2^	0.239 ***		0.298 ***		0.235 ***		0.353 ***		0.421 ***	
ΔR^2^	0.010 ^‡^^c^		0.066 ***				0.121 ***		0.068 ***	
*F-statistic* ΔR^2^	2.87 ^c^		22.98				15.14		28.47	

Notes: ^‡^ *p* < 0.10; * *p* < 0.05; ** *p* < 0.01; *** *p* < 0.001 References: ^a^ = female; ^b^ = egalitarian or not sure. Explained variance: ^c^ = ΔR^2^ for adding interaction term to Model 3.
